# Supplementary material for: Phenyllactic Acid as a Marker of Antibiotic-Induced Metabolic Activity of Nosocomial Strains of Klebsiella pneumoniae In Vitro Experiment
Source: Microorganisms. 2025 Nov 15;13(11):2599. doi: 10.3390/microorganisms13112599 (PMC12654857; doi:10.3390/microorganisms13112599)
Supplement: Supplementary file 1 [file microorganisms-13-02599-s001.zip › Supplementary File S1.pdf]

Supplementary S1 Clinical data on the resistance of hospital strains of K. pneumoniae isolated in this work

The table presents data on the resistance of hospital strains of *K. pneumoniae* to various antibiotics, obtained using standard methods accepted for work in this hospital.

Table S1. Evaluation of resistance of hospital strains of *K. pneumoniae* isolated in this work: R – resistance, I – intermediate sensitivity, S – sensitivity

**Antibiotics**	**Strains**									
	**A**	**B**	**C**	**D**	**E**	**F**	**G**	**H**	**I**	**J**
Amikacin	I	R	R	R	R	S	R	S	R	R
Imipenem	R	R	R	R	R	R	R	-*	R	-
Tigecycline	R	-	S	-	-	R	-	-	-	-
Cefepime	R	R	R	R	-	R	R	R	R	R
Ciprofloxacin	R		R	R	R	R	-	-	-	-
Meropenem	R	R	R	R	R	R	R	R	R	R
Ceftazidime	R		R	R	-	-	R	R	R	R
Ampicillin Sulbactam	-	-	-	-	-	-	R	R	R	-
Aztreonam	-	-	-	-	-	-	R	-	-	-
Colistin	-	-	-	-	-	-	S	S	S	R
Doripenem	-	-	-	-	-	-	R	R	R	-
Gentamicin	-	-	-	-	R	S	S	S	R	S
Levofloxacin	-	-	-	-	-	-	R	R	R	-
Piperacillin/Tazobactam	-	-	-	-	-	-	R	R	R	-
Ticarcillin/Clavulanic Acid	-	-	-	-	-	-	R	R	R	-
Tobramycin	-	-	-	-	-	-	R	S	R	-
Trimethoprim/Sulfamethoxazole	-	R	-	-	R	-	R	R	R	R
Amoxicillin/Clavulanic Acid	-	-	-	-	-	-	-	-	-	R
Ampicillin	-	-	-	-	-	-	-	-	-	R
Cefotaxime	-	-	-	-	-	-	-	-	-	R
Ciprofloxacin	-	-	-	-	-	-	-	-	-	R
Aztreonam	-	-	-	-	-	-	-	R	R	-

* – Due to the planned rotation of antibacterial drugs at the medical facility, some microbiological samples were not tested for susceptibility to certain antibiotics. It is important to note that this decision is made based on a thorough analysis of the epidemiological situation and taking into account recommendations for the rational use of antibacterial drugs.
