# Supplementary material for: Phenyllactic Acid as a Marker of Antibiotic-Induced Metabolic Activity of Nosocomial Strains of Klebsiella pneumoniae In Vitro Experiment
Source: Microorganisms. 2025 Nov 15;13(11):2599. doi: 10.3390/microorganisms13112599 (PMC12654857; doi:10.3390/microorganisms13112599)
Supplement: Supplementary file 1 [file microorganisms-13-02599-s001.zip › Supplementary File S2.pdf]

«УТВЕРЖДАЮ»
Директор Федерального бюджетного учреждения науки «Центральный научно-исследовательский институт эпидемиологии» Федеральной службы по надзору в сфере защиты прав потребителей и благополучия человека (ФБУН ЦНИИ Эпидемиологии Роспотребнадзора)

________________ В.Г. Акимкин
«22» 04 ________ 2019 г.

INSTRUCTIONS BY APPLICATION

DNA detection reagent kit enterobacteria, staphylococci And streptococci V biological material by the polymerase chain reaction (PCR) method
For diagnostics in vitro

"AmpliSense ® Florocoenosis / Aerobes - FL»

AmpliSense ®

ФБУН ЦНИИ Эпидемиологии
Роспотребнадзора,
Российская Федерация, 111123,
город Москва, улица Новогиреевская, дом 3А

IVD

CONTENT

LIST ABBREVIATIONS 3
PURPOSE 3
PRINCIPLE METHOD 4
FORMS EQUIPMENT 5
ANALYTICAL CHARACTERISTICS 6
MEASURES PRECAUTIONS AND INTELLIGENCE ABOUT DISPOSAL 8
ADDITIONAL MATERIALS AND EQUIPMENT 11
TAKE, TRANSPORTATION AND STORAGE SUBJECT MATERIAL 12
PREPARATION OF THE SUBJECT MATERIAL FOR EXTRACTION DNA 13
INTERFERING SUBSTANCES AND RESTRICTIONS ON THE USE OF SAMPLES SUBJECT MATERIAL 13
CONDUCTING PCR TESTS 15
EXTRACTION DNA FROM STUDY SAMPLES 15
FORM 1 (PCR kit option) FRT-100 F) 16
COMPOSITION 16
AMPLIFICATION WITH DETECTION IN MODE "REAL TIME" 16
A. Preparation samples For amplifications 16
B. Conducting amplifications With detection V mode "real time" 18
IN. Analysis And interpretation results 19
TERM TERMS OF USE. CONDITIONS TRANSPORTATION AND STORAGE 23
WARRANTY COMMITMENTS MANUFACTURER 23
SYMBOLS, USED IN PRINTED PRODUCTS 25
APPLICATION 1 26

LIST ABBREVIATIONS

The following abbreviations and designations are used in this instruction:

VKO- FL	- exogenous internal control sample
GE	- genomic equivalents
DNA	- deoxyribonucleic acid
dNTF	- deoxyribonucleoside triphosphate
STIs	- infections, transmitted sexual by way
K1, K2	- DNA calibrators
TO-	- negative control PCR
NK	- nucleic acids
OK	- negative control extractions
EYE	- negative control sample
PCR	- polymerase chain reaction
RU	- registration certificate
UDG	- Uracil DNA glycosylase
FBUN Central Research Institute Epidemiology of Rospotrebnadzor	- Federal budget institution sciences "Central Research Institute of Epidemiology" of the Federal Service for Supervision of Consumer Rights Protection consumers And well-being human
FRT	- fluorescent detection V mode "real time"

PURPOSE

Reagent kit **"AmpliSens ® FloroCenosis/Aerobes-FL"** is designed for the quantitative determination of DNA of enterobacteria (family *Enterobacteriaceae*), staphylococci (*Staphylococcus* spp.) and streptococci (*Streptococcus* spp.) in biological material (smear from the vaginal mucosa) using PCR with hybridization-fluorescence detection of amplification products in "real time" mode, in the diagnosis of infectious and inflammatory diseases of the urogenital tract of women.

The material for PCR is DNA samples extracted from the material being tested.

Indications and contraindications for the use of the reagent kit

The reagent kit is used in clinical laboratory diagnostics for the study of biological material obtained from women with suspected infectious and inflammatory diseases of the urogenital tract.

There are no contraindications, except in cases where material collection cannot be performed for medical reasons.

Potential users of medical products

Only medical workers trained in molecular diagnostic methods and rules for working in a clinical diagnostic laboratory are allowed to work with the reagent kit. V established in order (SP 1.3.2322- 08 "Safety of work with microorganisms of pathogenicity (hazard) groups III-IV and pathogens of parasitic diseases").

IN in accordance With federal by law from 21.11.2011 No. 323-FZ "On the Fundamentals of Health Protection of Citizens in the Russian Federation" PCR testing is one of the methods of comprehensive examination of a patient, on the basis of which the attending physician establishes a diagnosis and selects measures for treating the patient.

PRINCIPLE METHOD

The testing principle is based on DNA extraction from samples of the test material together with an exogenous internal control sample (VKO-FL) and simultaneous amplification of DNA sections of the detected microorganisms and VKO-FL DNA with hybridization-fluorescence detection. VKO-FL allows monitoring all stages of the PCR study for each sample and assessing the effect of inhibitors on the results of the PCR study.

The DNA samples obtained at the extraction stage are used to carry out a reaction to amplify a DNA region using primers specific to this region and the Taq polymerase enzyme. The reaction mixture contains fluorescently labeled oligonucleotides that hybridize with the complementary region of the amplified DNA target, resulting in an increase in fluorescence intensity. This allows the accumulation of a specific amplification product to be recorded by measuring the intensity of the fluorescent signal using an amplifier with a system detections fluorescent signal V mode "real time."

Quantitative determination of DNA of enterobacteria, staphylococci and streptococci is based on the existence of a linear relationship between the initial concentration DNA targets V under study sample And cycle

the beginning of the exponential increase in the fluorescent signal (threshold cycle, *Ct*). To conduct a quantitative test, DNA amplification from the samples under study is carried out simultaneously with DNA calibrators – samples with a known concentration of target DNA . Based on the results of amplification of DNA calibrators, a calibration line is constructed, along which the concentration of target DNA in the samples under study is determined.

The reagent kit contains a system for protection against contamination by amplicons by using the enzyme uracil-DNA glycosylase (UDG) and deoxyuridine triphosphate. The enzyme UDG recognizes and catalyzes the destruction of DNA chains containing deoxyuridine, but not DNA containing deoxythymidine. Deoxyuridine absent V natural DNA, but is always present in amplicons because deoxyuridine triphosphate is part of the dNTP mixture in the amplification reagents. Deoxyuridine makes contaminating amplicons susceptible to degradation by the enzyme UDG before amplification of the target DNA begins, and therefore they cannot be further amplified.

Enzyme UDG thermolabile And is inactivated at heating above 50 °C and therefore does not destroy the target amplicons produced during the PCR process.

At the amplification stage, 4 reactions are carried out simultaneously in one test tube – amplification of sections of enterobacteria, staphylococci and streptococci DNA, as well as amplification of the VKO-FL sequence. The results of amplification of enterobacteria, staphylococci and streptococci DNA, as well as VKO-FL DNA are recorded by 4 different fluorescent detection channels:

Table S2-1

**Channel for fluorophore**	**FAM**	**JOE**	**ROX**	**Cy5**
DNA target	*Enterobacteriaceae-aceae*	*Staphylococcus* spp.	*Streptococcus* spp.	DNA VKO-FL
Amplification region	*gene* 16S rRNA	*gene* 16S rRNA	*gene* 16S rRNA	artificial nucleotide sequence

FORMS EQUIPMENT

Form 1 includes a set of reagents "PCR-set" option FRT-100 F

Form 1 intended For conducting amplifications DNA with hybridization-fluorescence detection V mode
"real time" and allows for the detection of DNA in a quantitative format. To conduct a full PCR study, it is necessary to use a DNA extraction reagent kit.

Form 1 is designed to carry out 110 amplification reactions, including controls.

ANALYTICAL CHARACTERISTICS

The following specifications apply to this reagent kit :

Linear measurement range and analytical sensitivity (detection limit)

Table S2-2

Type of material being studied	Transport environment	DNA Extraction Kit	Amplification kit	Microorganism	Analytical sensitivity (limit detection), GE/ml [1]	Linear measurement range, GE/ml
Vaginal mucosal smears	"Transport medium with mucolytic (TSM)»	"DNA-sorb- AM»	PCR-set option FRT-100 F	*Enterobacteria-ceae*	$2x10^3$	$1x10^4$ – $1x10^8$
				Staphylococcus spp.		
				Streptococcus spp.		

These characteristic values are achieved by following the rules specified in the sections “Taking, transporting And storage under study material» And
"Preparation under study material To extractions DNA".

Analytical specificity

There were no non-specific reactions when testing the samples:

- DNA human V concentrations $5x10^7$ GE/ml;
- DNA of the following strains of microorganisms from the ATCC collection (American Type Culture Collection, USA) and clinical isolates at a concentration of $1x10^7$ GE/ml: *Lactobacillus* spp., *Gardnerella vaginalis* ATCC ® 14018 ™ , *Enterococcus faecium* ATCC ® 35667 ™ , *Neisseria gonorrhoeae* ATCC ® 49926 ™ , *Chlamydia trachomatis* , *Mycoplasma hominis* , *Ureaplasma urealyticum* , *Trichomonas vaginalis* , *Candida albicans* ATCC ® 14053, *HSV* 1 And 2 type (virus simple herpes types 1 And 2),

[1] The number of genomic equivalents of a microorganism (GE) in biological material (smear with mucous vagina), placed V specified transport Wednesday, V recalculated to 1 ml.

CMV (cytomegalovirus).

When testing DNA samples of microorganisms belonging to *Enterobacteriaceae* , *Staphylococcus* spp. and *Streptococcus* spp., including DNA of the following strains from the ATCC (American Type Culture Collection, USA) collection and clinical isolates at a concentration of at least 7x10 ^{7} GE/ml: *Escherichia coli* ATCC ® 25922 ™ *, Klebsiella pneumoniae* ATCC ® 27736 ™ *, Proteus mirabilis* ATCC® 12453TM , *Staphylococcus aureus* ATCC ® 6538P ™ *, Staphylococcus epidermidis* ATCC ® 12228 ™ *, Staphylococcus saprophyticus* ATCC ® 4990 ™ *, Streptococcus agalactiae* ATCC ® 12386 ™ *, Streptococcus pneumoniae* ATCC ® 49619 ™ , *Streptococcus pyogenes* ATCC ® 19615 ™ for each group, positive results were obtained only in the channel for detecting DNA of the corresponding group of microorganisms, and there were no non-specific results in other channels.

Information about interfering connections indicated V section "Interfering substances and restrictions on the use of samples of the test material."

Repeatability, reproducibility And right

Repeatability and reproducibility were determined by testing model biomaterial samples. Model biomaterial samples were prepared by diluting standard sample enterprises, containing DNA *of Enterobacteriaceae, Staphylococcus* spp. *, Streptococcus* spp. in concentrations of 1x10 ^{4} , 1x10 ^{6} and 1x10 ^{8} GE/ml in biological material – smears from the vaginal mucosa placed in a transport medium with mucolytic (TMM). To prepare model samples, pooled samples of smears from the vaginal mucosa were used, in which the DNA content of bacterial groups determined by the reagent kit did not exceed 1x10 ^{4} GE/ml. Repeatability conditions included testing in the same laboratory, by the same operator, using the same equipment within a short period of time. Reproducibility conditions – testing in different laboratories, by different operators, using different equipment.

Table S2-3

Repeatability

Microorganism	Initial value of concentrati on tions, GE/ml	Average number of repetitions	Average concentrati on value tions, lg	Mean standard deviation (SD)	Average coefficient of variation (CV), %
Enterobacteriaceae	$1x10^{4}$	20	4.35	0.15	3,52
Staphylococcus spp.		20	4,38	0,16	3,62
Streptococcus spp.		20	4,37	0,16	3,63
Enterobacteriaceae	$1x10^{6}$	20	6,40	0,05	0,81
Staphylococcus spp.		20	6,20	0,02	0,33
Streptococcus spp.		20	6,42	0.04	0.56
Enterobacteriaceae	$1x10^{8}$	20	8.33	0.18	2.18
Staphylococcus spp.		20	8.37	0.17	2.00
Streptococcus spp.		20	8.35	0.15	1.80

Table S2-4

Reproducibility

Microorganism	Original meaning concentratio ns , GE/ml	Average number of repetitions	Average value concentration s , lg	Standard average deviation (SD)	Average coefficient of variation (CV), %
Enterobacteriaceae	$1x10^{4}$	40	4.33	0.17	3.80
Staphylococcus spp.		40	4.35	0,16	3,72
Streptococcus spp.		40	4,35	0,16	3,69
Enterobacteriaceae	$1x10^{6}$	40	6,40	0,07	1,01
Staphylococcus spp.		40	6,20	0,04	0,71
Streptococcus spp.		40	6,42	0,05	0,72
Enterobacteriaceae	$1x10^{8}$	40	8.32	0.17	2.11
Staphylococcus spp.		40	8.36	0.17	1.99
Streptococcus spp.		40	8.36	0.14	1.68

Accuracy was determined by measuring the quantitative DNA content of the three identified bacterial groups in a dilution of the plant's standard sample at a concentration of $5x10^{4}$ GE/ml.

Table S2-5

Right

Microorganism	Average number of repetitions	Average measurement value, lg	Average set value	Average systematic error (B), %
Enterobacteriaceae	100	4.77	4.70	1.49
Staphylococcus spp.	100	4.68	4.72	0.83
Streptococcus spp.	100	4.66	4.70	1.57

PRECAUTIONS AND DISPOSAL INFORMATION

The work must be carried out in a laboratory that performs molecular biological (PCR) studies of biological material on availability pathogens

infectious diseases, in compliance with the sanitary and epidemiological rules SP 1.3.2322-08 "Safety of work with microorganisms of III-IV pathogenicity (hazard) groups and pathogens of parasitic diseases", SanPiN 2.1.7.2790-10 "Sanitary and epidemiological requirements for handling medical waste" and methodological guidelines of the MU 1.3.2569-09 “Organization of work of laboratories using nucleic acid amplification methods when working with material containing microorganisms of pathogenicity groups I-IV.”

At work necessary Always fulfill next requirements:

- The temperature in the laboratory room is from 20 to 28 °C, relative humidity from 15 to 75%.
- Consider the samples being studied as infectious and dangerous, organize work and storage in accordance with SP 1.3.2322-08 “Safety of work with microorganisms of pathogenicity (hazard) groups III-IV and pathogens of parasitic diseases.”
- Clean up and disinfect spilled samples using disinfectants means V in accordance with SP 1.3.2322-08 “Safety of work with microorganisms of pathogenicity (hazard) groups III-IV and pathogens of parasitic diseases.”
- The laboratory process should be one-directional. Analysis is being carried out V separate premises (zones). Work should begin in the Extraction Zone and continue in the Amplification and Detection Zone. Do not return samples, equipment, or reagents to the area where the previous step of the process was performed.
- Unused reagents, reagents with expired shelf life, as well as used reagents, packaging [2], biological material, including materials, instruments and objects contaminated with biological material, should be disposed of in accordance with the requirements of SanPiN 2.1.7.2790-10 "Sanitary and epidemiological requirements to the handling of medical waste."

ATTENTION! At removal waste after amplifications (tubes containing PCR products) must not be opened test tubes And splashing contents,

---

[2] Unused reagents, reagents With expired term suitability, The reagents used and the packaging belong to the medical waste hazard class G.

because This Maybe bring To contaminations products PCR laboratory area, equipment and reagents.

- Use and change disposable tips for automatic filter dispensers at each operation. Disposable plastic utensils (test tubes, tips) must be thrown into a special container containing a disinfectant that can be used to disinfect medical waste.
- Table surfaces, as well as the rooms in which the PCR is carried out, before and after completion
  works must be exposed to ultraviolet radiation for 30 minutes.
- The reagent kit is intended for single use to conduct PCR testing of the specified number of samples (see section “Composition”).
- The reagent kit is ready for use according to these instructions. Use the reagent kit strictly as intended.
- TO work With set reagents allowed only personnel trained in molecular diagnostic methods and rules for working in a clinical diagnostic laboratory in the established manner (SP 1.3.2322-08 "Safety of work with microorganisms of pathogenicity (hazard) groups III-IV and pathogens of parasitic diseases").
- Do not use the reagent kit if the inner packaging is damaged or if the reagent does not appear as described.
- Do not use the reagent kit if the transportation and storage conditions according to the instructions have not been observed.
- Do not use the reagent kit after the expiration date.
- Use disposable powder-free gloves, lab coats, and eye protection when working with samples and reagents. Wash your hands thoroughly after finishing work. All operations are carried out only with gloves. for exclusion contacts With organism human.
- Avoid inhalation of vapors, contact with skin, eyes and mucous membranes. Harmful if swallowed. On contact immediately rinse affected place water, seek medical attention if necessary.

- If the conditions of transportation, operation and storage are observed, there is no risk of explosion or fire.
- A reagent kit safety information letter is available upon request.

An assessment of the probable events that will result in negative consequences for the body may occur human.

When used as directed and the above precautions are observed, the reagent kit is safe.

Specific effects of the reagent kit on the body human:

- Carcinogenic effect absent.
- Mutagenic action absent.
- Reproductive toxicity absent.

ADDITIONAL MATERIALS AND EQUIPMENT

Take under study material

1. Transport medium - “Transport medium with mucolytic (TMM)” (RU No. FSR (2009/05514).
2. Probe gynecological universal (For example, ZGU "CM", LLC "CENTERMED", Russia (RU No. FSR 2011/11331).
3. A swab probe for collecting, transporting and storing biological samples (for example, DELTALAB SLU , Spain (RU No. FSR 2009/05516).

Extraction DNA from researched samples

4. A set of reagents for DNA extraction - "DNA-sorb-AM" (RU No. FSR 2007/00183).
5. Additional materials And equipment For DNA extraction – according to the instructions for the DNA extraction reagent kit.

Amplification with hybridization-fluorescence detection of amplification products

6. Disposable polypropylene test tubes at work With "PCR kit" option FRT-100 F:
   a) screw-top or tightly closed test tubes with a volume of 1.5 ml (for example, Axygen, Inc., USA, or similar) – for the preparation of the reaction mixtures.
   b) thin-walled PCR tubes with a volume of 0.2 ml and a convex or flat optically transparent lid

(e.g. Axygen, Inc., USA, or similar) or test tubes volume 0.2 ml V strips By
8 pcs. with transparent lids (for example, QIAGEN GmbH, Germany, or similar) – when using a tablet-type device;

c) thin-walled 0.2 ml PCR tubes with a flat cap (e.g., Axygen, Inc., USA, or similar) or 0.1 ml Rotor-Gene PCR tubes in strips of 4 with caps (e.g., QIAGEN GmbH, Germany, or similar) – when using a rotor-type device.

7. Disposable tips for variable volume pipettes with filter up to 100 µl (e.g. Axygen, Inc., USA, or similar).
8. Racks for 0.2 ml or 0.1 ml tubes (according to the tubes used) (e.g. Axygen, Inc., USA, or similar).
9. Boxing abacterial air Wednesday (PCR box) (For example, "BAV-PCR-"Laminar-S", JSC "Laminar systems", Russia, or similar).
10. Vortex (For example, SIA Biosan, Latvia, or similar).
11. Automatic variable volume dispensers (for example, Biokhit LLC, Russia, or similar).
12. A programmable amplifier with a real-time fluorescent signal detection system, having 5 or more independent channels fluorescent detections (For example, Rotor Gene Q (QIAGEN GmbH, Germany), CFX96 (Bio-Rad Laboratories, Inc., USA) .
13. Refrigerator from 2 to 8 °C with a freezer from minus 24 to minus 16 °C.
14. Separate gown, caps, shoes and disposable gloves according to MU 1.3.2569-09.
15. Capacity For reset tips.

TAKE, TRANSPORTATION AND STORAGE OF THE STUDY MATERIAL

Material For research serve smears with vaginal mucosa .

Smears with mucous vagina

Take material to conduct With with help tampon probe or universal probe V test tube With transport Wednesday from

posterolateral vaginal fornix. Move the working part of the probe along the surface of the lateral walls of the vagina with a rotating motion, collecting as much discharge as possible. Take a sufficient amount of material from the vagina. Minimal presence of impurities in the form of mucus and blood is acceptable. Transfer the probe to a test tube with a transport medium. Break off the working part of the probe containing the material to be examined and leave it in the test tube with the transport medium. If it is impossible to break off the working part of the probe, rinse the biomaterial from the working part into the test tube with the transport medium as completely as possible by pressing it to the inside of the test tube and rotating it 5-10 times clockwise and counterclockwise. Do not use scissors to cut the working part of the probe!

Close the tube tightly with a lid, avoiding gaps and crushing the inner part of the lid, and label it. When using a transport medium with a mucolytic, its color may change due to a change in pH (at an acidic pH of the mucous secretion).

It is permissible to store samples of material in a transport medium with a mucolytic (TMM) before conducting a PCR study:

- at temperature from 18 to 25 °C - V flow 28 days;
- at temperature from 2 to 8 °C - V flow 3 months;
- at temperature Not higher minus 20 °C - for a long time.

Single freezing and thawing of the material is allowed .

PREPARATION SUBJECT MATERIAL TO DNA EXTRACTION

Samples of smears from the vaginal mucosa do not require preliminary preparation.

INTERFERING SUBSTANCES AND RESTRICTIONS ON USE PROB SUBJECT MATERIAL

The influence of potentially interfering substances was studied on model samples. Model samples were prepared by diluting a standard sample enterprises containing DNA *of Enterobacteriaceae* , *Staphylococcus* spp., *Streptococcus* spp., *Streptococcus* spp. up to a concentration of 2x10 ^{3} and 2x10 ^{4} GE/ml in the OKO reagent (included in the reagent kit) For extractions DNA "DNA-sorb-AM") With with and without the addition of potentially interfering substances.

For ratings influences high concentrations exogenous

The following drugs were used as substances: Miramistin (Infamed, Russia) (reg. No. P N001926/01); Chlorhexidine bigluconate, 0.05% solution for external use (Biogen NPC, Russia); Clotrimazole, 1% cream (Ozon, Russia); Metrogil, vaginal gel (metronidazole 1%) (Unique Pharmaceutical Laboratories, India); Polygynax, capsules vaginal (Innotech International", France);
"Macmiror Complex", vaginal capsules (Polichem Srl, Italy); "Hasico For Women", lubricating gel ("EMANSI", Russia); "Contex Silk", intimate silicone lubricating gel (Contex, Czech Republic), "Play Feel", lubricating gel for increasing sensitivity (Durex, Thailand) in various concentrations (see Table S2-6). The concentration of the exogenous substance was determined as the volume of the preparation to the volume of the test sample. The maximum concentration of the exogenous substance corresponded to the maximum possible volume of the vaginal smear placed in the transport medium when collecting clinical material probes, recommended for use.

To assess the influence of endogenous substances, glycogen, hemoglobin, lactoferrin, and mucin were used in various concentrations. (cm. table. S2-6). Concentrations endogenous substances were determined taking into account the literature data on the study of PCR inhibitors. The highest concentration of endogenous substances exceeded the concentration of PCR-inhibiting substances by two times according to the literature data.

Table S2-6

**View potential interferent**	**Potential Interferent**	**Tested concentration**	**Presence of interference**
Endogenous substances	Hemoglobin	65; 130; 260 mcg/ml	Not found
	Lactoferrin	1.25; 2.5; 5 mcg/ml	Not found
	Glycogen	30; 60; 120 mg/ml	Not found
	Mucin	50; 100; 150 mcg/ml	Not found
Exogenous substances	"Miramistin"	4; 8; 16 % (volume of preparation to volume of the sample under study)	Not found
	"Chlorhexidine bigluconate"		Not found
	"Clotrimazole"		Not found
	"Metrogil"		Not found
	"Polygynax"		Not found
	"Macmiror complex"		Not found
	"Hasico For Women»		Not found
	«Contex Silk»		Not found
	"Play Feel»		Not found

The absence of influence of the studied potentially interfering substances is guaranteed only when using For extractions DNA set reagents "DNA-sorb-AM".

To control the efficiency of DNA extraction and the amplification reaction, the reagent kit provides for the use of an internal control sample (VKO-FL), which is added to each biological sample at the stage of nucleic acid extraction. At the end of the amplification reaction, the presence of a signal indicating the accumulation of VKO-FL DNA fragments indicates sufficient efficiency of nucleic acid extraction and the absence of PCR inhibitors.

CONDUCTING PCR RESEARCH

PCR test consists of from following stages:

- extraction DNA from samples under study,
- DNA amplification with hybridization-fluorescence detection in real time,
- analysis And interpretation results.

EXTRACTION DNA FROM STUDY SAMPLES

For extractions DNA is used set reagents "DNA-sorb-AM". Order works With complete set reagents For "DNA-sorb-AM", see the instructions for the extraction kit.

ATTENTION! When conducting a quantitative PCR study, it is unacceptable using a reagent kit "EDEM" And others express methods extractions DNA . Reagent and sample volumes for extraction using DNA-sorb-AM reagent kit:

DNA extraction from each test sample and controls is performed in the presence of an internal control sample – **VKO-FL** .

Volume **VKO-FL** – **10 µl** in each test tube. The volume of the test sample is **100 µl** .

IN test tube negative extraction control (OK) contribute **100 µl EYE** .

Volume elutions - **100 µl** .

FORM 1 ("PCR kit" option FRT-100 F) COMPOSITION

"PCR kit" option FRT-100 F – a set of reagents for amplification of DNA fragments of enterobacteria, staphylococci and streptococci with hybridization-fluorescence detection in the "real time" mode allows for PCR research in a quantitative format. The reagent set includes:

Reagent	*Description*	*Volume, ml*	*Quantity*
**PCR-mixture-FL FloroCenosis / Aerobes**	Transparent liquid from colorless to light purple	1,2	1 test tube
**PCR buffer- B**	Transparent colorless liquid	0.6	1 test tube
**Polymerase (TaqF)**	Transparent colorless liquid	0.06	1 test tube
**K–**	Transparent colorless liquid	0.2	1 test tube
**K1 AB**	Transparent colorless liquid	0.2	1 test tube
**K2 AB**	Transparent colorless liquid	0.2	1 test tube

The reagent kit is designed to carry out 110 amplification reactions, including controls.

AMPLIFICATION WITH DETECTION IN MODE "REAL TIME"

The choice of tubes for amplification depends on the amplifier used with a real-time detection system.

Disposable tips with filters are used to add reagents, DNA samples and control samples into test tubes.

A. Preparation samples For amplifications

The total volume of the reaction mixture is 25 µl, including the volume of the DNA sample – 10 µl.

ATTENTION! The components of the reaction mixture should be mixed immediately before the PCR study.

1. Mix content test tubes With reagents **PCR mixtures - FL Florocenosis / Aerobes , PCR buffers- B,**

polymerase (TaqF) and precipitate the droplets by short-term centrifugation (1-2 With) With with help vortex.

2. Preliminary necessary prepare mixture **PCR buffer-B** and **polymerase (TaqF)** . For this content one test tubes With **polymerase (TaqF) (60 µl)** must be completely transferred into a tube with **PCR buffer B (600 µl)** and mix gently on a vortex, preventing foam from forming. Label the test tube, indicating the date of preparation of the mixture.

ATTENTION! Prepared mixture calculated on study of **110** samples. The mixture should be stored at a temperature from 2 to 8 °C for 3 months and use as needed.

3. In a separate test tube, prepare the reaction mixture based on the consumption for one reaction (see Appendix 1):
   - **10 µl PCR-mixes-FL Florocoenosis / Aerobes** ,
   - **5 µl PCR-buffer-B** And **polymerases (TaqF).**
4. Select the required number of test tubes or strips for DNA amplification of the test and control samples.
5. **15 µl of the prepared mixture** to each test tube . Discard any unused remains of the reaction mixture.
6. Add **10 µl of DNA samples** obtained as a result of extraction from the samples being studied into prepared test tubes.

ATTENTION! When adding DNA samples extracted using the DNA-sorb-AM reagent kit, it is necessary to avoid getting the sorbent into the reaction mixture.

7. Put control reactions:

   A) **negative extraction control (NEC)** - add **10 to the test tube** with the reaction mixture **µl** of sample extracted from **EYE** .

   b) **negative PCR control (K–)** – add **10 µl of K–** to the test tube with the reaction mixture.

   V) **DNA calibrator K1** - V test tube With reactionary add the mixture **10 µl K1 AB.**

   G) **DNA calibrator K2** – V test tube With reactionary add the mixture **10 µl K2 AB.**

B. Conducting amplifications With detection V mode "real time"

1. Program the amplifier with a detection system in “real time” mode to perform the appropriate amplification program and detect the fluorescent signal (see Tables S2-7, S2-8) [3].

Table S2-7

Unified program for amplification and detection of fluorescent signal "AmpliSense" For rotary devices type [4] and tablet type [5]

Cycle	Temperature, °C	Time	Fluorescence detection signal By channels for fluorophores	Number of cycles
1	50	15 min	–	1
2	95	15 min	–	1
3	95	10 With	–	45
	60	20 With	**FAM, JOE, ROX, Cy5**	

ATTENTION! Using a single program, any combination of tests can be performed simultaneously in one device, including tests with reverse transcription and amplification. When several tests are performed simultaneously in the "multiprime" format, the detection of the fluorescent signal is also assigned to other channels used, in addition to those specified. In the event that tests are performed simultaneously in one device only for the detection of pathogen DNA, the first step of reverse transcription (50 °C – 15 minutes) to save time.

[3] Amplification programs (Tables S2-7, S2-8) are equivalent in use for this set of reagents.
[4] For example, Rotor Gene Q (QIAGEN).
[5] For example, CFX 96 (Bio- Rad) .

Table S2-8

Fluorescent amplification and detection program signal "AmpliSens-1"

Cycle	Devices rotary type [4]			Devices tablet type [5]		
	Temperature, °C	Time	Number of cycles	Temperature, °C	Time	Number of cycles
1	**95**	15 min	1	**95**	15 min	1
2	**95**	5 With	5	**95**	5 With	5
	**60**	20 With		**60**	20 With	
	**72**	15 With		**72**	15 with	
3	**95**	5 with	40	**95**	5 with	40
	**60**	20 with fluoresce detection. signal		**60**	30 with fluoresce detection. signal	
	**72**	15 with		**72**	15 with	

Detection of the fluorescent signal is assigned to channels for the FAM, JOE, ROX and Cy5 fluorophores.

Note – Channel For fluorophore **Cy5.5** turns on if necessary, If are being carried out tests V format "multiprime", For which is used this channel.

2. Place the tubes in the wells of the reaction module of the device. It is recommended to precipitate the drops from the walls of the tubes using a vortex before placing them in the plate-type amplifier.

ATTENTION! In case of incomplete loading of tablet-type devices, it is recommended to additionally install empty tubes along the edges of the reaction module of the amplifier.

3. Start the amplification program with fluorescent signal detection.
4. Once the program is complete, begin analyzing and interpreting the results.

IN. Analysis And interpretation results

The analysis of the obtained results is carried out using the software of the device used for PCR with detection in the "real time" mode. The curves of accumulation of the fluorescent signal are analyzed in 4 channels:

Table S2-9

Channel for fluorophore	FAM	JOE	ROX	Cy5
Registration signal indicating accumulation of product amplifications	DNA *Enterobacteriaceae*	DNA *Staphylococcus* spp.	DNA *Streptococcus* spp.	DNA VKO-FL

The results are interpreted based on the presence (or absence) of the intersection of the S-shaped (sigmoid) fluorescence curve with the threshold line set at the appropriate level, which determines the presence (or absence) of the threshold cycle (*Ct*) value for the given DNA sample in the corresponding column of the results table.

Based on the obtained values of the threshold cycle (*Ct*) and on the specified concentration values for the DNA calibrators K1 AB and K2 AB, a calibration line is automatically constructed and the number of copies of the target DNA of enterobacteria, staphylococci or streptococci is calculated for the test and control samples.

ATTENTION! Meanings concentrations DNA calibrators indicated in insert, attached To set reagents.

The obtained values are used to calculate the number of genomic equivalents of DNA *of* the corresponding microorganisms contained in 1 ml of the original sample of biological material, according to the formula:

[Число копий] ДНК микроорганизмов x K = [Число геномных эквивалентов] на 1 мл (ГЭ/мл)

ATTENTION! The coefficient **K** for calculating the result in GE/ml is indicated in the insert included with the reagent kit.

The calculated values of the concentrations of DNA of enterobacteria, staphylococci and streptococci reflect the total content of these microorganisms in the test material placed in the transport medium.

If the obtained value is less than $1x10^4$ GE/ml, then the result “less than $1x10^4$ GE/ml” is indicated if the obtained value is more than $1x10^8$ GE/ml, then the result “more than $1x10^8$ GE/ml” is indicated (taking into account the linear range of the kit).

The analysis result **is invalid** if the *Ct value* for the Cy5 fluorophore channel for a given sample is absent or is greater than the limit, while the calculated values of the concentrations of enterobacteria, staphylococci or DNA streptococci Not defined or make up less 10 ^{4} GE/ml. Repeated PCR testing of this sample is required, starting from the DNA extraction stage.

DNA of enterobacteria, staphylococci and streptococci is not detected if the *Ct value* for enterobacteria DNA for a given sample is staphylococci or streptococci is absent, and the *Ct value for the channel for the Cy5 fluorophore is determined* to be less than the limit.

ATTENTION! The limit values are indicated in the insert included with the reagent kit .

The result of the PCR test is considered reliable, if correct results are obtained for the DNA extraction and amplification stage controls in accordance with Table S2-10 and the insert included with the reagent kit.

Table S2-10

Results For controls various stages PCR research

Control	Controlled stage PCR-research	Results amplifications By channel For fluorophore			
		FAM	JOE	ROX	Cy5
OK	DNA extraction	concentration value is missing or defined less borderline	concentration value is missing or defined less borderline	concentration value absent or defined less than the boundary	value defined *Ct* is less than the limit
TO-	PCR	concentration value is missing or defined less borderline	concentration value is missing or defined less borderline	concentration value absent or defined less than the boundary	meaning *Ct* is missing
K1	PCR	value defined *Ct*	value defined *Ct*	value defined *Ct*	value defined *Ct*
K2	PCR	*Ct* value is determined to be less than borderline	*Ct* value is determined to be less than borderline	*Ct* value is determined to be less than borderline	defined O *Ct* value is less borderline

Possible errors:

1. For DNA calibrators (K1, K2), the threshold cycle (*Ct*) values for the channels for the FAM and/or JOE and/or ROX fluorophores are missing, or the *Ct value* for DNA calibrator K2 exceeds the cutoff value, or the efficiency index **E** according to the standards graph is less than the value specified in the package insert. It is necessary to repeat the amplification and detection for all samples.
2. For the negative extraction control (NEC) and/or the negative PCR control (K-), the calculated concentration value (in GE/ml) of enterobacteria and/or staphylococci and/or streptococci DNA exceeds the limit value specified in the package insert. It is necessary to repeat the PCR test for all samples, starting with the DNA extraction stage.

TERM FITNESS. TERMS AND CONDITIONS TRANSPORTATION AND STORAGE

Shelf life. 12 months. The reagent kit with an expired shelf life is not subject to use. The shelf life of opened reagents corresponds to the shelf life indicated on the labels for unopened reagents, unless otherwise specified in the instructions.

Transportation. Kit reagents transport at temperature from 2 to 8 °C for no more than 5 days in thermal containers containing cooling elements, by all types of covered vehicles. Transportation is permitted at temperatures from 2 to 25 °C for no more than 3 days. "PCR kit" option FRT-100 F upon receipt, disassemble in accordance with the specified storage temperatures.

Storage. "PCR kit" option FRT-100 F keep V refrigeration camera at temperature from 2 to 8 °C, except for PCR buffer B and polymerase (TaqF). PCR buffer B and polymerase (TaqF) should be stored in a freezer at a temperature from minus 24 to minus 16 ° C. PCR mixture-FL FloroCenosis / Aerobes store in a place protected from light.

Refrigeration and freezing chambers must ensure a regulated temperature regime.

WARRANTY COMMITMENTS MANUFACTURER

The manufacturer guarantees that the main parameters and characteristics of the reagent kit comply with the requirements specified in the technical and operational documentation during the specified shelf life, subject to all conditions of transportation, storage and use.

The consumer is prohibited from using components from different series of reagent kits.

The medical device is not subject to technical maintenance and repair.

Complaints about the quality of the reagent kit should be sent to the address: 111123, Moscow, Novogireevskaya St., Building 3A, e-mail: cs@pcr.ru [6]

If side effects not specified in the instructions are detected By application set reagents, unwanted

---

[6] Reviews and Suggestions about AmpliSens products you you can leave, having filled consumer questionnaire on the website: www.amplisens.ru .

reactions during its use, facts and circumstances that pose a threat to the life and health of citizens and medical workers during the use and operation of the reagent kit, it is recommended to send a message to the address indicated above and to the authorized state regulatory organization (in the Russian Federation – Federal Service for Surveillance in Healthcare) in accordance with current legislation.

Заведующий НПЛ ОМДиЭ
ФБУН ЦНИИ Эпидемиологии
Роспотребнадзора

Е.Н. Родионова

Главный врач ФГБУ «Поликлиника № 1»
УДП РФ

Е.В.Ржевская

SYMBOLS, USED IN PRINTED PRODUCTS

REF	Number By catalog		Caution! Refer to the instructions By application
LOT	Code parties	Σ	Contents enough to conduct n- number of tests
IVD	Medical device for diagnostics *in vitro*		Use to
VER	Date changes	i	Contact us To instructions by application
	Temperature range		Not admit exposure to sunlight
	Manufacturer		Date manufacturing

APPLICATION 1
Scheme preparations reactionary mixtures

	Volume reagents on specified quantity reactions, µl	
**Volume reagents on one reaction, µl**	**10.0**	**5.0**
**Number of biological samples studied [7]**	**PCR-mixture-FL FloroCenosis / Aerobes [7]**	**Mixture PCR buffer-B and polymerases (TaqF)**
1	60	30
2	70	35
3	80	40
4	90	45
5	100	50
6	110	55
7	120	60
8	130	65
9	140	70
10	150	75
11	160	80
12	170	85
13	180	90
14	190	95
15	200	100
16	210	105
17	220	110
18	230	115
19	240	120
20	250	125
21	260	130
22	270	135
23	280	140
24	290	145
25	300	150
30	350	175

[7] Provided meanings With taking into account stock (calculation on one reaction more) And With taking into account the need to set up 4 control reactions: K1, K2, OK and K-.
