# Supplementary material for: Phenyllactic Acid as a Marker of Antibiotic-Induced Metabolic Activity of Nosocomial Strains of Klebsiella pneumoniae In Vitro Experiment
Source: Microorganisms. 2025 Nov 15;13(11):2599. doi: 10.3390/microorganisms13112599 (PMC12654857; doi:10.3390/microorganisms13112599)
Supplement: Supplementary file 1 [file microorganisms-13-02599-s001.zip › Supplementary File S3.pdf]

Supplementary S3. PhLA production for hospital strains of K.pneumoniae after incubation in the presence of different antibiotics.

Table S3. PhLA production (µmol/l) for hospital strains of K.pneumoniae (n=10) after incubation in the presence of different antibiotics. PhLA values, µmol/l, corresponding to the concentration in the control tube without antibiotics are marked in blue, values exceeding the control value are marked in red, PhLA values that do not differ significantly (within ±20%) from the control are marked in yellow, and values significantly lower than the control are marked in green. R – resistance, I – intermediate sensitivity, S – sensitivity, measured by the standard method

**Antibiotics** / **strains**	PhLA, µmol/l/antibiotic resistance									
	A	B	C	D	E	F	G	H	I	J
Doxycycline	0,0	6,6	0,0	-0,4	1,0	13,1	4,3	-0,2	0,1	2,4
Nitrofurantoin	0,3	0,7	2,6	1,8	4,6	6,3	3,1	0,2	0,0	1,9
Rifampicin	-0,1	2,6	0,5	4,7	8,1	11,8	6,4	2,7	0,6	2,8
Clarithromycin	0,1	8,2	7,4	11,2	9,5	31,0	6,1	5,1	2,3	7,9
Trimethoprim/sulfamethoxazole	-/-	-/-	-/-	10,5	10,2	20,2	6,9	6,3	2,8	7,3
Meropenem	1,0/R	37,7/R	12,4/R	13,3/R	13,5/R	30,1/R	19,7/R	5,8/R	4,5/R	12,0/R
Cefepime	- /R	- /R	- /R	10,0/R	11,4/-	22,6/R	9,5/R	5,0/R	2,9/R	- /R
Cefotaxime	-/-	-/-	-/-	10,1	10,5	24,5	8,6	5,4	2,5	- /R
Ciprofloxacin	1,2/R	10,9/-	7,9/R	9,8/R	10,3/R	28,7/R	11,2/-	4,8/-	3,1/-	9,5/-
Tigecycline	0,0/R	0,1/-	0,0/S	7,3/-	0,2/-	17,9/R	6,2/-	-0,2/-	-0,1/-	0,0/-
Amikacin	0,0/I	7,6/R	7,5/R	11,8/R	9,5/R	6,8/S	15,3/R	5,6/R	2,7/R	5,6/R
Imipenem	- /R	16,9/R	12,9/R	15,5/R	31,5/R	21,0/R	2,1/R	4,8/-	1,3/R	3,2/-
Ceftriaxone	1,2	10,9	8,3	-/-	-/-	-/-	-/-	-/-	-/-	9,7
Ceftazidime	- /R	10,3/-	5,5/R	- /R	- /R	-/-	-/-	- /R	- /R	9,0/R
K. Pneumoniae	1,1	12,2	6,8	12,2	7,4	24,4	8,8	5,2	2,3	6,9
